# Active Gaze Control Improves Optic Flow-Based Segmentation and Steering

**DOI:** 10.1371/journal.pone.0038446

**Published:** 2012-06-14

**Authors:** Florian Raudies, Ennio Mingolla, Heiko Neumann

**Affiliations:** 1 Center of Excellence for Learning in Education, Science, and Technology (CELEST), Boston University, Boston, Massachusetts, United States of America; 2 Center for Computational Neuroscience and Neural Technology (CompNet), Boston University, Boston, Massachusetts, United States of America; 3 Institute for Neural Information Processing, University of Ulm, Ulm, Germany; University of Muenster, Germany

## Abstract

An observer traversing an environment actively relocates gaze to fixate objects. Evidence suggests that gaze is frequently directed toward the center of an object considered as target but more likely toward the edges of an object that appears as an obstacle. We suggest that this difference in gaze might be motivated by specific patterns of optic flow that are generated by either fixating the center or edge of an object. To support our suggestion we derive an analytical model that shows: Tangentially fixating the outer surface of an obstacle leads to strong flow discontinuities that can be used for flow-based segmentation. Fixation of the target center while gaze and heading are locked without head-, body-, or eye-rotations gives rise to a symmetric expansion flow with its center at the point being approached, which facilitates steering toward a target. We conclude that gaze control incorporates ecological constraints to improve the robustness of steering and collision avoidance by actively generating flows appropriate to solve the task.

## Introduction

In the 20^th^ century research on optic flow revealed its invariant properties and influence on steering control. This early research defined optic flow as the apparent change of structured light in the optic array over time and shows that this optic flow is useful to segment a visual scene [1] or to control aircrafts [2]. Decades later, experiments showed that humans can judge heading within two degrees of accuracy from random dot kinematograms that were designed to generate optic flow cues only [3]. Human heading accuracy depends on dot density and observer speed [4]. Simulated eye-movements in these displays increase errors in estimated heading, and errors are biased in the direction of the simulated rotation in the optic flow [5], [6]. Heading judgments for the same displays are robust with respect to simulated noise or limited dot lifetime for either translational or rotational motion above a ground-plane or through a 3D dot cloud [7]. Experiments in virtual environments show that optic flow is used for controlling walking toward a target in conjunction with pure positional information about the target [8]. In sum, optic flow provides information about heading, and humans use this information to control their behavior.

Active gaze control, or an active vision approach, can help to solve inverse problems in vision. For instance, ill-posed problems such as reconstructing shape from shading, from contour, or from texture become well-posed under appropriate active control of the camera [9], [10]. Another example is the estimation of optic flow from image sequences that becomes well-posed when employing active vision strategies [9]. In theory, many ill-posed inverse problems in vision become well-posed or better constrained by assuming an active vision approach.

**Figure 1:**
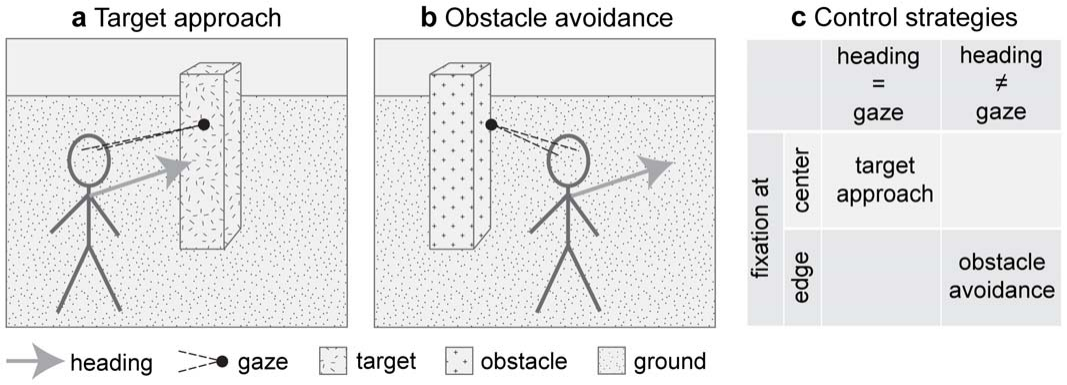
Illustration of two tasks that generate two strategies for gaze control. **a)** If humans approach a target they fixate the center and align heading with gaze. **b)** To avoid an obstacle the outer edge is fixated and gaze points to a direction that is different from the direction of heading. This outer edge is an apical edge. These two sketches are based on the findings reported by Rothkopf & Ballard [14] and Fajen & Warren [15]. **c)** These strategies are embedded in a larger set of possible control strategies that depend on gaze and heading, the point of fixation, and the role of the faced object to be either target or obstacle. The control strategies from a) and b) are used by humans. Our derived model provides a flow-based analysis of all four possible strategies that does not include the detection of an object to be target or obstacle, e.g. by a color cue as in the experiment.

Eye-tracking data shows that active gaze control is used by humans. Gaze is actively controlled in ordinary daily tasks [11], e.g. the making of a cup of tea [12] or steering a vehicle on the road during driving [13]. When preparing a peanut butter and jelly sandwich, humans deploy an orchestrated sequence of eye-movements that are astonishingly similar between participants when given the same abstract instruction [11]. Eye-tracking data shows that humans are actively selecting gaze points that are related to the current task [11], [12]. During a visual navigation task humans fixate the center of targets while they prefer to fixate the edge of obstacles [14]. An illustration is shown in Figure 1. What generates this task-dependent difference in gaze behavior? Why do we not use one of the other possible control strategies? For instance we could fixate the center of an obstacle or the edge of a target, as shown in Figure 1c.

We suggest an optic-flow based explanation for the gaze behavior during visual steering. This explanation is based on a combination of eye-tracking data and walking trajectories for the same tasks [15]. We develop an analytical model and run simulations for the detection of flow discontinuities and flow patterns in these tasks of visual navigation. This model explains patterns of gaze and heading for target approach and obstacle avoidance on the basis of local and global flow cues. We make two claims based on the model analysis. (i) For flow-based steering control the point being approached can be directly extracted from flow, if the observer proceeds in the same direction. This occurs if the observer is close to the target. In this case heading and gaze are aligned, and fixating eye−/head-rotations and body rotations are small and can be neglected. This translational motion can be identified due to zero-valued curl and shear components and a positive divergence component of the flow. (ii) Local flow derivatives are maximized if a wall that either is planar or smoothly curved is tangentially fixated. This could facilitate flow-based segmentation.

Our flow-based explanation makes the following assumptions: (i) that no independently moving objects occur, (ii) that the object for fixation is identified and (iii) that the scene is rigid. Rigid means that the scene contains no deformable objects, e.g. a face that is changing its emotional expressions. Note that we do not require the segmentation of the edge or object from the background. Instead, we offer a flow-based explanation for such a segmentation that is a search for discontinuities in the flow. Furthermore, once the segmentation is achieved our analysis provides information about controlling gaze in order to maintain fixation at the edge which can be facilitated by the detection of flow-based discontinuities.

Our analysis includes visual cues in form of optic flow that is generated from parameters of gaze and heading for target approach or obstacle avoidance. Mathematical descriptions of flow for a sensor moving with a translational and rotational velocity through a rigid environment [16] are used to derive a model for the spatially local flow derivatives assuming various edge types, such as fixating tangentially a planar or curved surface, or an apical edge. Tangential fixation is defined by looking parallel to an object’s surface. An apical edge is characterized by a continuous transition on one side of the edge and an abrupt change on the other side. For instance, in Figure 1b fixation is directed to an apical edge of the object.

**Figure 2:**
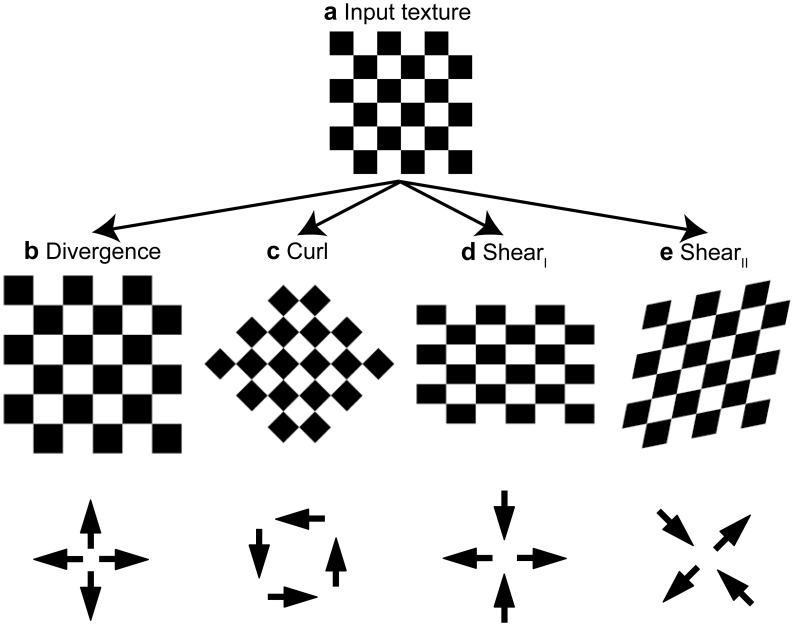
Illustration of the decomposition of flow into divergence, curl, and shear components. **a)** Shows the input pattern, a regular checkerboard texture. Subsequent panels show the resulting deformed image for a divergence in **b)**, a curl in **c)**, a Type I shear in **d)** and a Type II shear in **e)**, and all of these components are positive. For illustration purposes these deformations were applied to a regular texture pattern. Later we will look for these deformations locally in image sequences whose spatio-temporal gray-value changes can be described by image flow models from the introduction. The depiction is adapted from Koenderink [19], his Figure 7. These figures depict divergence, curl, and shear applied to texture patterns. There is also an interpretation of these components for global flow patterns (see text in the Introduction).

*Local* flow derivatives can be decomposed into geometrical components of divergence, curl, and shear as suggested by Koenderink and van Doorn [17] and Braddick [18]. Figure 2 shows an example of such decomposition. Assume a regular checkerboard texture that is transformed by flow. This flow can be described by its divergence, curl, and shear components. These components are applied separately to the input texture in Figure 2a. Applying divergence gives the texture pattern shown in Figure 2b, applying curl the texture in 2c, applying Type I shear the texture in 2d, and applying Type II shear the texture in 2e. Note that for Type II shear the axis for the frame of reference is rotated by 45° compared to the reference frame of Type I shear.

These flow derivatives have also an interpretation with respect to patterns of *global* flow defined for the entire visual field. In such an interpretation, (i) divergence relates to an expansional global flow pattern that is a result of forward motion. The opposite interpretation holds for contractive global flow patterns. (ii) A global flow pattern that originates from rotating the sensor around the optical axis gives rise to curl. Type I shear or Type II shear do not exclusively or directly relate to one single type of self-motion.

**Figure 3:**
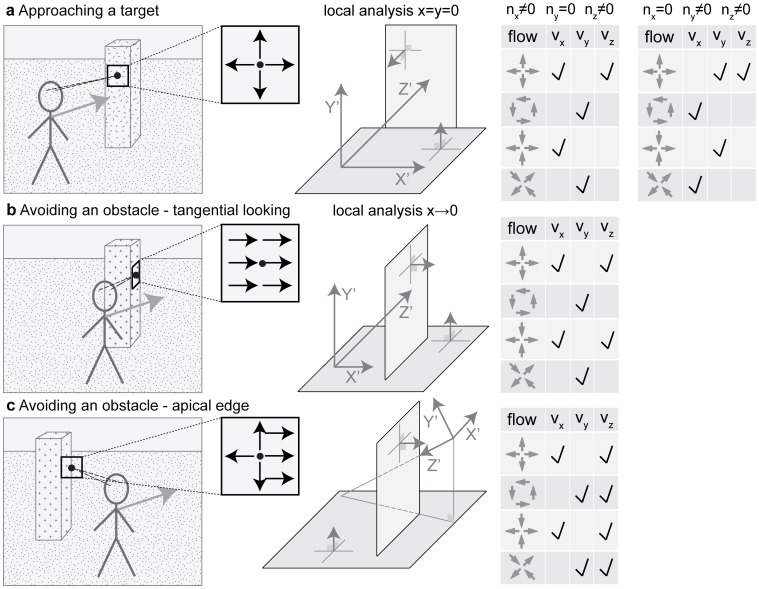
Summary of the local flow derivative analysis. **a)** Approaching a target while fixating the surface *n_x_* ≠ 0, *n_y_* = 0, and *n_z_*≠0 yields divergence and Type I shear components if moving sideward; curl and Type II shear components if moving up/downward; a divergence component if moving forward, compare with the Table 1. For the case *n_x_* = 0, *n_y_* ≠ 0, and *n_z_* ≠ 0 the same components occur if swapping *x* and *y*, compare with the Table 2. **b)** Avoiding an obstacle by tangentially fixating its edge surface yields the same flow derivative components as in a) for the case *n_x_* = 1, *n_y_* = 0, and *n_z_* = 0. **c)** Fixation of an apical edge yields all four components if moving along the optical axis. Sideward movement results in divergence and Type I shear and up−/downward movement in curl and Type II shear components.

Our work analyzes flow derivatives that occur for different forms of self-motion and gaze direction. Figure 3 provides an outline and overview of this idea. On the left-hand side of the figure three scenarios are displayed: fixation at the center of a target object, fixation parallel to the edge of an obstacle, and fixation at the outer edge of an obstacle called an apical edge. The first column shows drawings of these 3D scenarios of facing a plane, looking tangential to a plane, and looking at an outer edge that has the background on the right side, in this example. The last column of Figure 3 shows the different flow derivative components of divergence, curl, and shear that occur in these configurations. These derivative components are characterized by the normal vector of the surface with components *n_x_*, *n_y_*, and *n_z_*, and the translational self-motion vector with components *v_x_*, *v_y_*, and *v_z_*. Note that flow derivatives are encoded using the same icons as in Figure 2. According to our derived analytical model some types of derivatives only occur for a specific configuration. For instance, in the target approach in Figure 3a with *n_x_*≠0, *n_y_* = 0, and *n_z_*≠0 and if the observer translates only along the x-axis, only divergence and Type I shear occur. See Figure 3a the table in the first row and there the entries for *v_x_*. More details about the analytical model and its comparison to responses from a biologically motivated flow derivative detector are given next.

**Figure 4:**
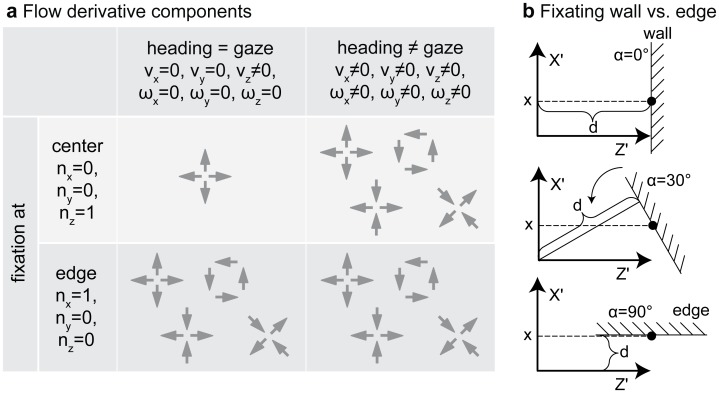
Shows the expected flow derivative components and the limit case applied to approximate flow derivatives at depth discontinuities. **a**) Repeats the table from Figure 1 showing the expected flow derivative components. For the target approach strategy only a divergence component is present that is the case in the upper right with fixating the center and alignment between heading and gaze direction. Note that not all cases could be distinguished by their qualitative pattern response. Therefore, we study the quantitative response at discontinuities like an edge. **b**) Shows the approximation used for an edge. The first graphics depicts fixation of a plane orthogonal to gaze direction. In the two succeeding graphics the wall is rotated by 90° until fixation appears tangential to the gaze direction.

**Figure 5:**
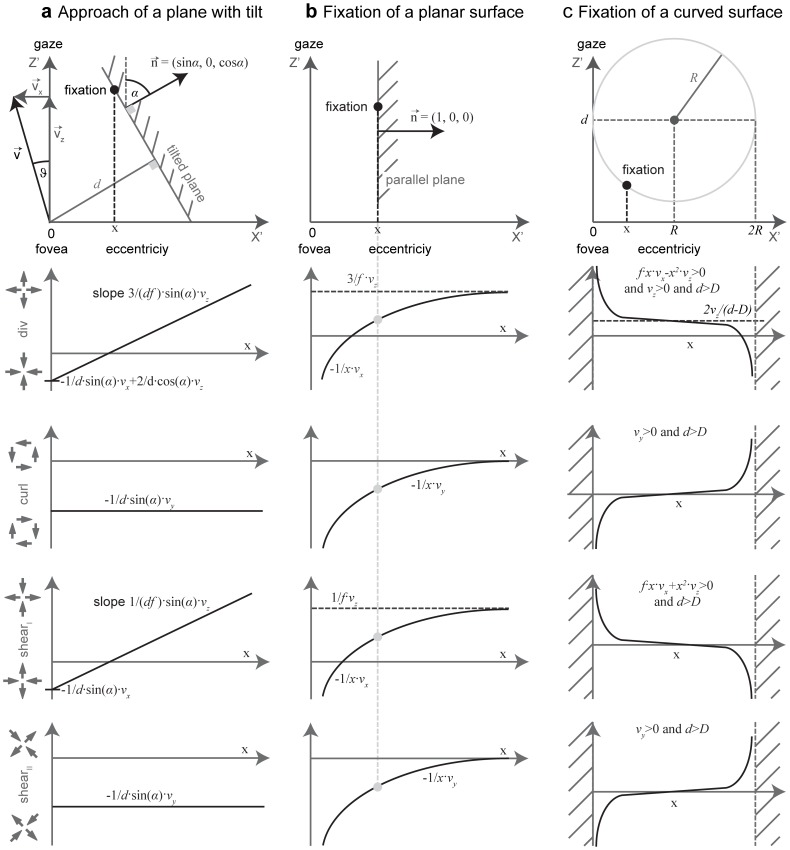
Response for flow divergence, curl, and shear get infinitely large if fixating tangentially. These strong responses located at objects’ edges allow for their segmentation. In all cases the camera has the translational velocity *(v_x_, v_y_, v_z_)*. **a)** Sketch of the scenario for fixating a plane that has a tilt *α* >0 in the *xz*-plane. The lower four rows show the functions for the response of flow components depending on the declination from the center of the visual field along the horizontal axis. Divergence and Type I shear depend linearly on *x*, the declination along the *x*-axis. Curl and Type II shear are independent of *x*. **b)** For a plane parallel to the optical axis all four components are reciprocal dependent on *x*. For values close to zero the component’s responses approach minus infinity. In this panel we depicted div, curl, and shear components by the gray-dashed line for a specific eccentricity or horizontal distance *x* of the plane from the gaze direction. **c)** A curved surface that is cylindrical in the *xz*-plane and planar in the *xy*/*yz*-planes leads to the same response characteristics for divergence, curl, and shear. For *x*→0 or *x*→*R* all four components approach ±infinity. The difference in linear versus hyperbolic response curves for case a) versus b) and c) gives an explanation why segmentation of obstacles can be improved by fixating the edge. Such a fixation results in strong responses that could be detected by difference operators. All sketches in the first row show a top-down view of the three scenarios, respectively.

## Results

In the following paragraphs we support our two claims by stating results for our derived model of flow discontinuities. We also include simulations using biologically inspired mechanisms for the detection of flow derivatives and their interconnection to provide responses for divergence, curl, and shear. First, we give possible interpretations for the finding by Rothkopf & Ballard [14] that fixation goes to an object’s center in a target approach task, because the generated flow pattern encodes the point of approach and simplifies steering control. Second, we analytically describe the fixation of obstacle edges by flow discontinuities which can be used for localization of an obstacle in the visual field. Third, we analyze flows extracted from videos and compute divergence, curl, and shear responses using the biologically inspired mechanisms to illustrate our developed mechanisms for generated and recorded videos.

### Fixating Targets at Their Center while Aligning Gaze and Heading

The fixation of a target’s center together with the alignment of gaze and heading has two implications for the generated flow fields that can contribute to the solution of the task of approaching a target. First, if gaze and heading are aligned, a translational flow results that is, ideally, a symmetric pattern of flow vectors. Within each sector, flow vectors point away from the point of fixation. This flow has a divergence component of 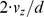. Curl and shear are zero, see Figure 4a. This argument excludes any rotations either originating from the eye, head, or body and supports our first claim.

**Table 1:** Shows the decomposition of a flow field into responses of divergence, curl, and shear.

Sensor	Scenario	div	curl	shear_I_	shear_II_	row
	Tilted plane	/	−	/	−	1
Pinhole	Parallel plane	∩	∩	∩	∩	2
	Parallel circle	∩	∩	∩	∩	3
	Tilted plane	−	0	−	0	4
Monopole periphery	Parallel plane	∩	0	∩	0	5
	Parallel circle	∩	0	∩	0	6
	Tilted plane	/	/	−	−	7
Monopole fovea	Parallel plane	∩	∩	∩	∩	8
	Parallel circle	∩	∩	∩	∩	9

The meaning of symbols is: “/” response depends linear on the declination *x* that is the distance to the center of the visual field measured along the x-axis; “−” constant response independent of *x*; “∩” response with hyperbolic dependence on *x*, a discontinuity is attained if fixating the edge tangentially; “0″ no response. The cortical transform changes the different response characteristics; however, for all three sensors the tilted plane does not result in a hyperbolic response curve. Thus, fixating at the center (tilted cases) is qualitatively different from fixating at the edge (parallel cases).

**Table 2:** Shows a decomposition of the Jacobian matrix for different self-motions.

	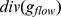	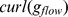	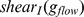	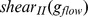
Translation	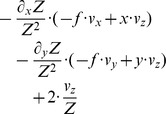	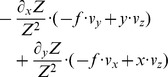	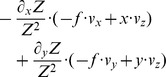	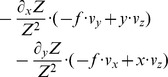
Rotation^1^	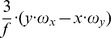	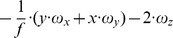	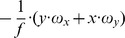	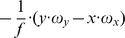
Fixating rotation	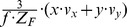	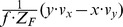	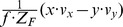	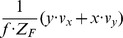
Translation toward a plane^2^	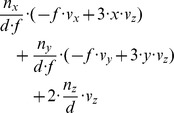	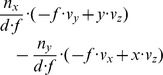	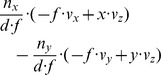	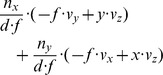

This matrix *J_flow_* is decomposed into divergence, curl, Type I shear, and Type II shear using the function *g_flow_* considering only a translation *(v_x_, v_y_, v_z_)* (second row), only a rotation *(ω_x_, ω_y_, ω_z_)* (third row), only a fixating rotation (fourth row), or a translational motion toward a plane with the normal *(n_x_, n_y_, n_z_)* and distance *d* (fifth row). Note, 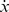 and 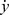 are the differential displacements on the image plane or pixel velocities. Rotations are independent of the depth and its partial derivatives.

1 The roll rotation *ω_z_* is independent of the surface. Furthermore, this roll rotation appears only and exclusively in the curl component.

2 The divergence 2•*v_z_/Z* is present, independent of the spatial derivatives of the depth *Z* and this component only appears for the divergence.

From a behavioral point of view excluding rotations makes sense, since such rotations would shift the center of motion (COM) and this shifted COM does not correspond to the theoretical focus of expansion (FOE). Coming closer to the target, human subjects show a reduced amount of body rotation [15]. In combination with the eye-tracking data [14] this suggests small eye-rotations and no head rotations while fixating the target’s center. Together, these data suggests that gaze and heading direction are aligned and that eye and head rotations are small as an observer comes close to a target.

Second, if gaze and heading direction are aligned, then the location of the FOE coincides with the point being approached when assuming a constant self-motion. Humans could use the detection of the FOE to align their walking direction with their gaze in order to accomplish straight walking and, furthermore, to align both with the target’s center in order to navigate toward the target. After successful alignments the target center, heading, and gaze coincide and all point toward the point being approached. We have thus supported our first claim that fixating the center of an object and aligning gaze, heading, and the point being approached supports steering toward the target; furthermore, only a positive flow divergence results. The second claim about flow-based segmentation namely, that fixating the edge facilitates a flow-based obstacle segmentation, will be supported next.

### Fixating the Obstacle’s Edges while Avoiding a Collision with the Obstacle

In order to avoid an obstacle it has to be visually segmented from the background. In addition to texture, color, and stereo cues, flow fields can provide a cue for segmentation. But what should the pattern of self-motion and gaze be to generate flow that best facilitates segmentation? Qualitative flow derivative components caused by different patterns of self-motion and gaze are shown in Figure 4a. Fixating the edge of an object, the derivative components that are present are the same regardless of the alignment between gaze and heading direction. Thus, a more qualitative analysis might be helpful. In order to approximate flow derivatives at an edge, we apply two steps; see Figure 4b. First, the edge is rotated until it is parallel to the direction of gaze. Second, the distance between optical axis and the plane that describes the edge is moved toward zero in the limit. Figure 5 and Table 1 provide qualitative values for flow derivatives. In general, directing gaze toward the center of an obstacle leads to smaller divergence, curl, and shear components than fixating the obstacle’s surface tangentially or fixating one of the obstacle’s outer or apical edges. These depictions are based on equations given in Table 2 for a general surface function *Z* and in Table 3 replacing the general surface function *Z* with a plane. Equations from Table 3 show that fixating the center of an obstacle leads to responses that are linear for varying *x*, the distance of the fixation point from the surface, or these responses have a constant slope for varying *x* (see 1^st^ column in Figure 5). This is unlike the response curves from column two and three in Figure 5 which are hyperbolic. The singularity of these curves is reached in the limit case of tangential fixation of a planar (2^nd^ column in Figure 5) or curved surface (3^rd^ column in Figure 5). For tangential fixation the infinitely large values of divergence, curl, and shear could be used to first establish tangential fixation and second to segment objects from the background as they occur at this transition. This finding is also shown in Table 1 using a compact notation. Fixating the center leads to straight curves (2^nd^ row in Table 1), and tangential fixation gives hyperbolic response curves for planar (3^rd^ row) and curved surfaces (4^th^ row) and thus shows the second claim from the introduction. These hyperbolic are directly linked to discontinuities in depth: “In the velocity field they [depth discontinuities] appear also as singular curves where the expansion, vorticity [curl] and shear take on infinite values”, cited from page 54 in [19].

**Table 3:** Shows the decomposition of the Jacobian matrix for fixation of a tilted surface and tangential fixation of a surface.

Fixating the center of an object surface that is tilted w.r.t. gaze				
	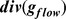	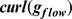	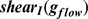	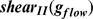
Plane tilted by *α* with respect to gaze^1^	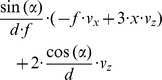	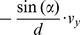	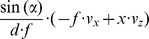	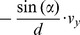
**Fixating the edge of an object surface tangentially**				
	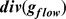	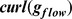	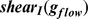	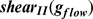
Plane parallel to gaze^2^	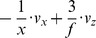	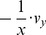	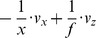	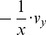
Curved edge^3^	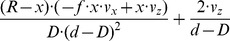	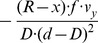	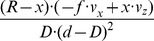	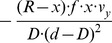

This decomposition uses a pinhole camera model and we assume *y = *0.

1 The tilt angle *α* is defined between the plane’s normal vector and the direction of gaze.

2 In this case the plane is parallel to gaze and appears at a very small distance of *x* aside from the gaze. Note that in the limit, where the distance approaches zero, the derivative components approach ±infinity.

3 In this case a circle is defined with the radius *R* at the position *(X, Z)*  =  *(R, d)*. The discriminant is 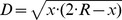.

To illustrate the derived properties we chose the following example: A forward motion 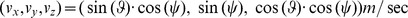 with 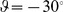 and 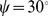, the distance 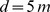 for the object 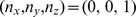, and the focal length of the pinhole camera is 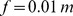. Fixating the center of the object gives a flow divergence of 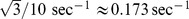 in the fovea locally around the fixation point with 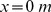 and 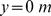. In this case the curl and shear components have a zero value. In contrast, the fixation of the rim of an object results in strong components of divergence, curl, and shear. Assume the location of the derivative to be computed at 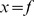 away from the fixation point, because at the fixation point the derivatives are not defined. For this case the divergence is 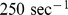, the curl is 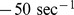, the Type I shear is 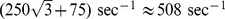, and the Type II shear is 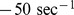. Curl and Type II shear have the same values, compare also with Table 3 for the case 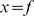. The components for a *fixating eye-rotation* are comparatively small (compare with the third row in Table 1 and set 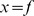, 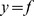, and 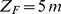, e.g. the divergence is 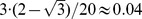 sec^-1^) and were neglected in the above analysis.

Flow derivatives for a curved edge are approximated by assuming tangential fixation of a circular post. We assume again 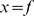 and 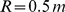. In this case the divergence is 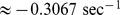, the curl is 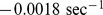, the Type I shear is 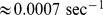, and the Type II shear is 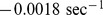. Again curl and Type II shear components are identical, compare with Table 2. Compared to the tangential fixation of a plane these components are small; however, in their limit case of *x* approaching zero they also approach ±infinity. These previous examples show that tangentially fixating the edge of a planar surface leads to strong divergence, curl, and shear components, which are much stronger than those generated by fixating the center of an obstacle approximated by a plane orthogonal to the gaze direction. In addition these components of tangential fixation are also much stronger than those generated by a fixating eye-rotation. Components of tangential fixation on a planar surface are stronger than those for tangential fixation on a circular post, assuming the same distance *x*  =  *f*.

So far, the analysis of divergence, curl, and shear in the flow fields refers to the image plane of a pinhole camera model. To study the same expressions in a representation as in the primary visual cortex in primates we use an additional monopole mapping [20] that models the cortical magnification for the transform of visual space as a function of polar coordinates to cortex. This monopole mapping is applied before computing partial derivatives to define the Jacobian matrix and then divergence, curl, and shear in the new coordinate system (see Appendix S1). For this representation we study the response characteristics for the periphery (the limit 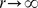) and for the fovea (the limit 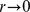). Qualitative results for these two regions in the visual field are given in Table 1; the lower two groups labeled by ‘Monopole periphery’ and ‘Monopole fovea’. For both regions within the visual field, response curves are linear in declination *x* for the fixation of a tilted plane (4^th^ row and 7^th^ row in Table 1). In contrast, the tangential fixation leads to hyperbolic responses (5^th^, 6^th^, 8^th^, and 9^th^ row in Table 1). Strong divergence, curl, and shear components can be used to better segment an obstacle from the background. The same applies if using foveal vision modeled by a monopole mapping.

### Simulation of Divergence, Curl, and Shear Components for Video Sequences

**Figure 6:**
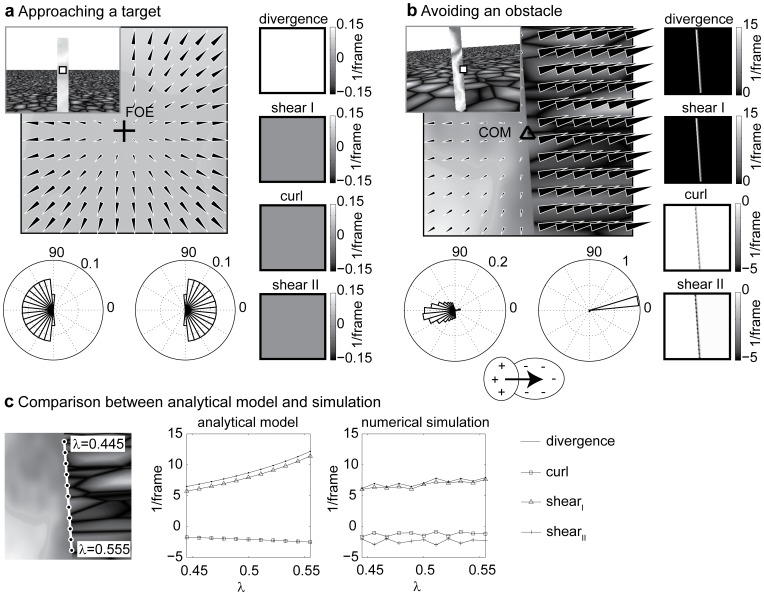
Display of flow and flow derivative components for the central five degrees of the visual field. **a)** If approaching a target, the flow has a focus of expansion (FOE) that marks the point being approached assuming constant motion. The overlaid image shows the scene with a 50 degrees horizontal field of view with a small square denoting the foveal five degrees of the visual field. The two circular histograms show the angular distribution of the motion direction for the circular left and right half of the visual field. Applying the antagonistic center-surround operator to compute the partial derivatives results in the divergence, curl, and shear components depicted in the four right panels. The shade of gray encodes the response strength, lighter encodes positive responses and darker encodes negative responses. Note this flow has only a positive divergence encoded by white; the other components are zero encoded as gray. **b)** When avoiding an obstacle it is fixated at the edge while the observer is walking around it. The resulting flow of this self-motion has a center of motion (COM) that does not coincide with the theoretical FOE due to the rotational components for fixation. Angular distributions of flow peak approx. 180 degrees apart and thus fit optimally to the derivative operator shown under the histograms. The flow field shows a strong discontinuity between object and background indicated by the strong divergence, curl, and shear components. Divergence and Type I shear show positive responses encoded as white stripes. Curl and Type II shear have negative responses encoded as black stripes. Note that the flow for the fixation of a cylindrical pillar shows the same qualitative behavior and is thus not shown. **c)** Comparison between the analytical model and the simulation. The left panel displays the input image superimposed with the model of an edge line where *λ* is within the range depicted in the white boxes. In the center panel curves for divergence, curl, and shear components are presented for samples along the edge line top to bottom and using the analytical model of spatial flow. The graph in the right panel shows the same edge samples that are numerically computed from a discretized image sequence. Samples are from pixels that are nearest neighbors to the points shown in the first panel. Due to the approximation of partial derivatives by biologically inspired operators and approximations in the apical edge model, values differ between model and simulation. In this plot evaluations of the analytical model are scaled by a value of 30 in order to match the simulations results.

After developing our theoretical model we analyzed video sequences in terms of their flow-based divergence, curl, and shear components. Synthetic videos were generated using the configurations of Rothkopf & Ballard [14]. Figure 6 shows results of this simulation (details about parameters etc. are given in the Method Section). Analytical flow for the central five degrees of visual field (horizontally and vertically) is computed and depicted by black arrows. The angular distribution of motions in the circular left and right half of the visual field is shown in the circular histograms. For the target approach both halves of the visual field show a distribution of angles over 180°. In contrast, the obstacle avoidance with the fixation strategy leads to narrow distributions approximately 180° apart. This distribution is optimally suited to be picked up by the biologically inspired operator as depicted below the histograms in Figure 6b. The type of filter and its visual representation has been taken from Born & Bradley [21] their Figure 6C. Their study provided evidence that motion discontinuities are detected by orientation selective filter mechanisms with juxtaposed ON and OFF subfields. Such filter sensitivities have been measured in half of the population tested, see Xia et al. [22]. Results for spatial flow derivatives that are computed by using such operators and their decomposition into divergence, curl, and shear components are displayed in the small panels on the left in Figure 6a and 6b. For an approaching observer only the divergence component is strictly positive over the entire field of representation. All other components are zero. This matches the case of purely expansion flow that is shown in Figure 6a; where the FOE of this flow indicates the point being approached that is directed to the center of the target object. In contrast the flow for a fixating observer has more variability. Fixation at the edge gives a discontinuity in depth in the field of view that leads to mainly translational flow for the foreground object. This is visible at the center of motion that is positioned at the obstacle's edge in Figure 6b. In the background the flow pattern is dominated by rotational flow, since the translation flow components are only minor in magnitude due to their large distance from the observer (compare also with Equations (2)). This mainly translational flow on the object and the mainly rotational flow on the ground are very different. The translational flow component is expansional while the rotational flow component is laminar. At the boundary of these two regions a strong discontinuity in the flow direction and speed occurs. See again in Figure 6b the zoomed-in image. This discontinuity leads to strong components of divergence, curl, and shear that are suited to segment the object from the background. In sum, the foveation in the target approach strategy gives expansion flow with the FOE at the point being approached while the fixation in the obstacle avoidance strategy results in a mixture of translational and rotational flow with a strong discontinuity in motion direction and speed at the location of the obstacle’s edge.

In order to characterize these component responses we derived an analytical model for the divergence, curl, and shear components of a moving observer. Thus, the responses of Figure 6a and 6b could be explained by our theoretical model (see Table 2 and Table 3 in the methods section). For the scenario in Figure 6a the plane is fixated with the gaze direction orthogonal to the plane’s surface, thus *(n_x_, n_y_, n_z_)* = *(0,0,1)*. Independent of the angle between the direction of gaze and heading, only a positive divergence component of 2•*v_z_/d* is present that encodes an expansion flow (or source); all other components are zero. The value of this divergence component depends inversely on the distance of the plane and is proportional to the translational speed along gaze.

Divergence, curl, and shear components for the scenario in Figure 5b can be approximated by using Equations (10a–c) from the methods section. Plugging in the values for this scenario given in the methods section into Equation (10a–c) gives the graph for the locations sampled from the edge line depicted in Figure 6c. The corresponding values of the simulation are shown in Figure 6c – center and right panel. While the curves do not exactly match each other their ordinal relationship is preserved and the main trend of the responses is the same. Major differences occur between the curl and Type II shear components. The analytical model gives identical responses, while they differ in the simulation. These differences might be explained by the approximation *∂_y_Z*≈0 in the model that was used in Equation (9) in the methods section, the approximation of difference operators, and the discrete sampling of the surface.

### Initial Segmentation by Finding Extreme Values for Flow Derivatives

**Figure 7:**
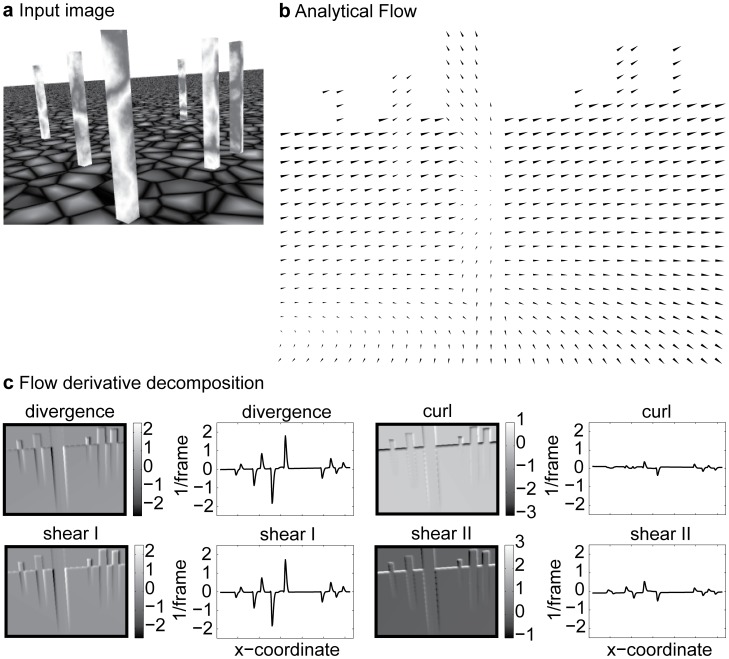
Shows the flow-based segmentation of obstacles for a horizontal 50° field of view with no monopole mapping. **a)** First frame of two with 320 × 640 pixels. **b)** Analytical flow for translational self-motion while fixating the point at the center of the visual field. **c)** Decomposition of flow derivatives into divergence, curl, and shear. Graph plots show the profile from the center row. Flow derivative components are largest for the fixated obstacles and could be used to identify and segment it against the background.

An additional simulation used a horizontal 50° field of view in order to compare derivative components of the fixated object against background and other objects. Figure 7a shows the 1^st^ image frame and Figure 7b the analytical flow that was used to compute the partial derivatives by applying the biologically motivated mechanisms (see methods section). Derivative components are strongest for the fixated obstacle assuming that it is the closest object (see Figure 7c). The strongest components, here divergence and Type I shear, can be used to segment the fixated closest obstacle from the background and other potential obstacles. If all obstacles are completely visible from a first-person perspective, responses of opponent sign can be grouped into a single object. This excludes configurations in which obstacles overlap or are only partly visible within the visual field.

**Figure 8:**
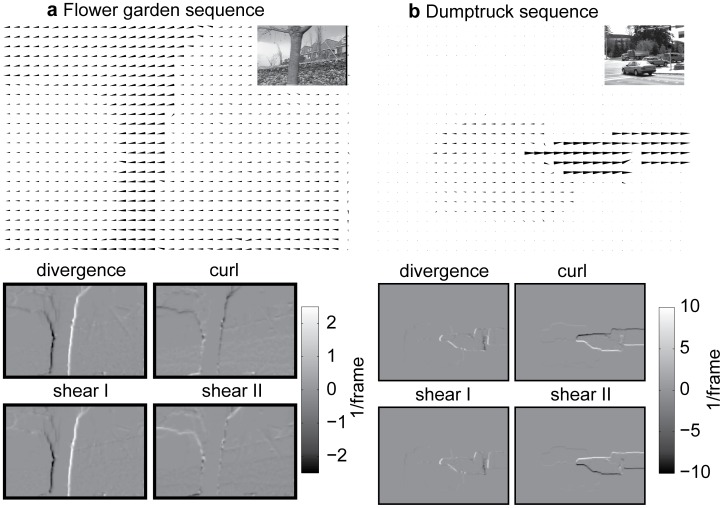
Computation of flow and derivative components for video sequences. **a)** Flow sampled by every 10^th^ value is depicted by arrows for the transition of the 15^th^ to the 16^th^ frame (counting from zero) of the ‘Flower garden’ sequence. The inset shows the 15^th^ image frame. The four panels depict divergence, curl, and shear. Between foreground and background strong divergence and Type I shear responses occur. According to the theory (Equations 10a–c) only these components occur for a pure sideward translation at a depth discontinuity. **b)** Flow sampled by every 20^th^ value for the frame transition 10^th^ to 11^th^ frame of the ‘Dumptruck’ sequence. The 10^th^ image frame is shown as inset. Four panels depict divergence, curl, and shear components. For these object motions mainly curl and Type II shear responses occur.

In order to generalize flow-based scene segmentation further, we apply the biologically inspired mechanisms to flow detected from video sequences. We choose two representative video sequences, one sequence consisting of self-motion and the other of independent object motion. In the self-motion sequence ‘Flower garden’ [23] the camera is arranged as if looking out of a side window from a driving car. The object motion sequence ‘Dumptruck’ [24] shows a typical traffic scenario of moving cars at an intersection (see insets in Figure 8a and 8b). Both sequences are considered as passive viewing, since the camera is not actively maintaining gaze at a fixation point. Flow was computed using the algorithm of Brox et al. [25]. The computed flow is shown in Figure 8a for the ‘Flower garden’ sequence and in Figure 8b for the ‘Dumptruck’ sequence. Strong divergence and Type I shear components occur at the boundaries of the tree that is in front of the flower garden, very similar as in the scenario depicted in Figure 7c. Derivative components for 'Flower garden' are depicted in the lower panels of Figure 8a. Strong divergence and Type I shear components fit to the theoretical model of an apical edge. For a sideward translation only divergence and shear Type I components occur at a depth discontinuity, compare with Equations (10a–c) in the methods section and set *v_x_*≠0, *v_y_* = 0, and *v_z_* = 0. For object motions in the 'Dumptruck' sequence strong curl and Type II shear components are present, see Figure 8b lower panel. Note that none of the cases in our analytical model describe object motions. This simulation shows that flow discontinuities occur at depth discontinuities or the transition of independent object motions and in both cases these discontinuities can serve as a cue for flow-based object segmentation.

## Discussion

This work derived an analytical model for spatial flow derivatives decomposed into divergence, curl, and shear and simulated biologically inspired derivative operators to compute these flow derivatives for passive and active viewing. We related analytical and simulation results to each other, and discussed how our model relates to behavioral data on human visual steering and gaze direction. Our analytical model provides a description of flow discontinuities for a pinhole camera and a monopole mapping that depend on scene and motion parameters. Using our model we showed that if heading and gaze are aligned only a flow divergence component occurs (see Figure 4) and the focus of expansion indicates the point being approached. Flow discontinuities respond maximally for tangentially fixated planar or smoothly curved surfaces (see Table 1). A summary of the different local flow derivatives decomposed into divergence, curl, and shear components for edge types and velocities is shown in Figure 3a for target approach and in Figure 3b and 3c for obstacle avoidance. Note that entries in each of the tables shown in Figure 3c combine linearly. This allows for several predictions that are summarized in Table 4. For target approach we expect a linear dependency of flow divergence, curl, and shear regarding the distance from the fovea. In contrast, for obstacle avoidance, either for tangential fixation or fixation of an apical edge, flow divergence, curl, and shear approaches ±infinity in the limit. For a finite sampling schema we expect large values for derivatives. Note that this qualitative difference of flow derivatives between target approach and obstacle avoidance is independent of the self-motion and depends only on the surface properties next to the point of fixation. Model and simulation results give a flow-based explanation for gaze points in visual navigation tasks of obstacle avoidance and target approach. This explanation could be seen as an example of affordance-based guidance of behavior based on optic flow in task-dependent visual navigation [26].

**Table 4:** Shows the decomposition of a flow field for target approach and obstacle avoidance.

**Configuration**	**Flow**			
	div	curl	shear_I_	shear_II_
**Target approach** (n_x_≠0, n_y_ = 0, n_z_≠0)				
v_x_ sideward motion	/	0	/	0
v_y_ up/down motion	0	/	0	/
v_z_ forward/backward motion	/	/	/	/
**Target approach** (n_x_ = 0, n_y_≠0, n_z_≠0)				
v_x_ sideward motion	0	/	0	/
v_y_ up/down motion	/	0	/	0
v_z_ forward/backward motion	/	/	/	/
**Obstacle avoidance** (tangential fixation)				
v_x_ sideward motion	∩	0	∩	0
v_y_ up/down motion	0	∩	0	∩
v_z_ forward/backward motion	∩	∩	∩	∩
**Obstacle avoidance** (apical edge)				
v_x_ sideward motion	∩	0	∩	0
v_y_ up/down motion	0	∩	0	∩
v_z_ forward/backward motion	∩	∩	∩	∩

Regardless of the self-motion target approach leads to linear responses indicated by “/” and obstacle avoidance to hyperbolic responses indicated by “∩”. Note that the meaning of the symbols is the same as in Table 1. A tangential fixation of a surface leads to qualitatively the same flow derivatives as fixation of an apical edge.

A discussion of prior theoretical work and the relation to our model and simulations follows below. We discuss fixating self-motion, flow statistics, prior work on the decomposition of flow derivatives and scene-constraint models of flow.

*Self-motion estimation* is a challenging problem. The estimation of self-motion based on optic flow is commonly formalized as a non-linear estimation problem using Longuet-Higgins & Prazdny’s [16] model for optic flow. For certain scene types the estimation of self-motion from optic flow has multiple solutions [27]. In other words, different self-motions and scene types lead to identical flows for the model of Longuet-Higgins & Prazdny. To simplify or constrain the problem of estimating self-motion we argue that humans use an active vision approach by selecting a specific gaze pattern combined with a particular self-motion pattern that leads to a trajectory and a sequence of scene views that are useful for solving the task of avoiding an obstacle or approaching a target.

*Fixating self-motion* simplifies various problems related to the estimation of flow, self-motion, and time-to-contact. Applying a fixation constraint for the estimation of self-motion has been shown to reduce the dimensionality of the search space from three parameters to one parameter using a flow template matching algorithm based on normal flow [28]. This requires the tracking of a point by rotational motions, the estimation of self-motion, and time-to-contact from the spatio-temporal derivatives of the image intensity function. Such a computation is a variant of a direct method that estimates self-motion directly from image intensities [29]. An alternative simplification projects spherical flow onto a longitudinal and latitudinal unit vector on the sphere, which reduces the computation of self-motion and time-to-contact to two 1D searches along meridians of the sphere [30]. A similar approach analyzes the pattern of flow signs on the sphere to estimate parameters of self-motion [31]. The same idea of pattern analysis has also been presented for the pinhole camera model in the context of robust self-motion estimation from normal flow [32]. In contrast to the computation of self-motion, we focused on the different characteristics of flows for behavioral strategies of approaching a target or avoiding an obstacle. While the approach situation is well analyzed in the literature in terms of estimating self-motion and relative depth (or time-to-contact), fixating self-motion has been studied only from the perspective of flow and self-motion estimation [33], [34] and self-motion estimation using a monopole mapping [35].

Our analysis provides a model for recent behavioral findings of visual navigation in humans. Rothkopf & Ballard [14] show data where humans fixate around the center of a target object that they approach and next to the edge of an obstacle object that they avoid. Their study used an eye-tracking and position tracking system, the latter to update the participant’s position in a virtual environment that is displayed through video goggles. Fajen & Warren [15] used the same experimental setup without the eye-tracking. Their experiment measured participants’ trajectories for navigation tasks of obstacle avoidance and target approach. Recorded trajectories show only small rotations for a target approach as the subject is close to the target. Combining the results from both studies suggests that humans during target approach keep their gaze and heading aligned, while they fixate the center of the target object. Our theoretical analysis shows that in this case the FOE is aligned with the point being approached and a control strategy simply has to align this FOE with the desired target location. For obstacle avoidance gaze and heading are independently controlled. Gaze is directed toward the edge of the obstacle, while heading is not necessarily aligned with gaze. Our explanation from the flow analysis suggests that subjects direct their gaze toward the edge to enhance the detection of the flow discontinuity and, thus, the localization of the obstacle. Tangential fixation of an edge leads in the limit to infinitely large responses that should be easily registered. Overall our analysis suggests a flow-based explanation for recent behavioral data of visual navigation tasks.

*Alternative hypotheses* for visual navigation focus on the image statistic and route selection. Rothkopf and Ballard [14] propose that gaze is not purely controlled by bottom-up image characteristics, like contrast, simple or complex cell responses, but also influenced by the task in a top-down manner. Their analysis of the foveated image shows strong responses for vertical edges in case of obstacle avoidance and no strong responses for neither vertical nor horizontal edges for target approach. Fajen and Warren [15] suggest that “route selection may emerge from online steering dynamics, making explicit path-planning unnecessary” (page 343). In their suggested steering dynamics obstacles act as repellent forces and goal objects act as attractive forces. Both types of forces control a second order dynamics (damped spring type model) to control the steering yaw-angle. Their model uses a birds-eye view to define repellent and attractive forces. In contrast we suggest a first-person perspective view. This alternative view allows for the flow-based segmentation of objects from the background. Estimates of object positions in the image can be integrated into dynamics similar to Fajen and Warren’s to explain walking paths [36], [37].

Another line of research looked at the *statistics of flow fields* for a moving camera sensor. Spatial and temporal statistics of image sequences recovered from a hand-held camera and car-mounted camera have been analyzed to form a prior for flow estimation [38]. Gradient-based, energy-based, and correlation based methods often show a very consistent bias for the estimated flow toward slower speeds and toward the major flow direction measured in a local neighborhood. A bias correction would be possible, if the noise characteristics were known; however, this is unlikely for a changing environment. Thus, the bias has been described as a property intrinsic to flow estimation and linked to visual illusions [39]. Retinal flow speed is tightly coupled to the scenes’ depth statistics. The direction of flow vectors in the image plane is tightly coupled to the direction of heading with respect to gaze (compare also with Equations 1 and 2). These couplings depend on the position in the visual field [40]. Another example of characterizing the statistics of flow fields used movies recorded for a person walking in natural environments. The data from the flow analysis shows that oblique motion directions have a broader and more asymmetric distribution of motion energy compared to cardinal directions [41]. All these analyses provided statistical measures of flow computed from different image materials. But results were not linked to the underlying flow model of self-motion from Equation (1) and (2). The other way around, methods for self-motion estimation use the statistics of self-motion flows [42], [43], [44]. In contrast, we suggest a model of the foveal flow statistics during self-motion for target approach and obstacle avoidance. We suggest that this foveal flow statistics is generated by subjects on purpose and in accordance to the task by actively controlling their gaze and heading.

The detection of singularities and *local flow analysis* has a long history. It ranges from first qualitative descriptions [1], [2], [18] to quantitative model analyses [17], [45], [46]. These analyses point out: The inverse relationship of flow to translational speed, compare with Equation (1), and the generation of motion parallax at depth discontinuities [47]. For instance, local flow divergence has been linked to time to contact estimation and obstacle avoidance for technical systems [48]. Recently, it has been formally shown that for a spherical camera model the point of the maximum divergence always appears halfway of the arc between the translational direction and surface normal. This maximum divergence can be estimated without knowing translational direction or surface normal. This makes the maximum divergence point suitable for steering control [49]. A complete decomposition of flow derivatives into divergence, curl, and shear components has been derived by Koenderink and van Doorn [17]. In this context complete means changing the basis vectors for the representation of local flow derivatives as applied in Equation (5). The new basis is defined by divergence, curl, and two shear components. Koenderink and van Doorn’s description was limited to a translational motion of the observer above a plane and used spherical coordinates for a spherical camera model. Nevertheless, their Equations (17–19) [17] bear some similarity to the equations presented in Table 3 in the methods section. Note that our work continues with this local flow analysis, but builds upon the more general flow model of Longuet-Higgins and Prazdny [16]. Table 1 specifies these same derivatives (for divergence and curl) in terms of a general surface function *Z(x,y)* and a general self-motion that contains translations and fixating rotations. As special cases we analyzed planes, edges tangentially viewed with and without curvature, and an apical edge. We analyzed flow represented at the level of primary visual cortex assuming a monopole mapping [50]. Details are given in Tables 5 and 6 in the Appendix S1. This analysis emphasizes that singularities are events that can be robustly detected in flow fields [51].

A local decomposition of flows or, in general, *higher order spatial flow derivatives* has been used for flow estimation. An approximation of temporal changes in the intensity function uses non-linear terms including higher order flow derivatives. These terms are embedded into a linear least-squares problem to estimate flow together with various flow derivatives in order to describe the intensity change more precisely [52]. In another context, flow on the cortical sheet has been defined by using a dipole mapping. Such a representation has been shown to be beneficial as a representation to estimate translational self-motion and roll-rotation when using a Helmholtz decomposition of cortical flow into a divergence free, a curl free, and a harmonic vector field [53] (chapter 7). The same Helmholtz decomposition has been embedded into a variational calculus to provide the regularization for flows from non-rigid structures that include high divergence and curl components, e.g. fluid motion [54]. In sum, higher-order flow derivatives have been used to gather additional constraints [52], to simplify the estimation by a dipole mapping of flow with high divergence and curl components [53] (chapter 7), or to specify further regularization for specific flow types [54].

*A constraint flow model for scene segmentation* uses the assumption that the scene can be approximated by a tessellation of multiple planes of varying and unknown distance and orientation. Assuming sampling from a plane by plugging the constraint from Equation (6) into the flow Equation (1) results in second-order polynomials for 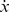 and 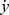 in arguments *x* and *y*. The coefficients of these polynomials depend entirely on the parameters of self-motion and the parameters of the plane. All these parameters are constant either over the entire visual field or for each planar surface within the visual field. Thus, a voting strategy can be used to segment the scene into planar surfaces of different motion and scene parameters [55]. Such an approach builds upon flow being compatible with a model for a specific parameterization within the entire visual field or the projected image region of a planar surface patch. In contrast, our approach explicitly characterizes the flow discontinuity as it appears for edges and emphasizes on the importance of its detection. Thus, voting uses the piecewise compatibility whereas our approach uses the incompatibility of flows at depth discontinuities. These discontinuities have been used to segment a foreground object from the background for a moving observer (Figure 8a) or independently moving objects for a stationary observer (Figure 8b).

The development of a real-world application must focus on closing the loop between extracting sensory information and motor steering control. We focused on the extraction of flow-information and gaze/heading strategies that control the flow information. An application would have to solve the selection of fixation points that we excluded from this study since it requires not only bottom-up but also top-down context information [56].

In summary, we introduced an analytical model for the decomposition of flow derivatives into divergence, curl, and shear components considering a model of fixating self-motion. Based on the analytical model and simulations we showed that fixation of an obstacle edge results in strong flow derivative components that could be used to (i) segment the object and (ii) to maintain fixation at the edge. This could explain why humans fixate the edge because of exploiting the actively generated flow to improve segmentation using eye rotations. For a target approach the strategy is the opposite. At a close distance toward the target eyes, head, and body do not rotate and fixation is directed toward the center of the target object. This allows for the association of the focus of expansion with the point being approached. In conclusion, optic flow is a rich source for segmentation and steering control and might explain the difference in gaze patterns for humans: fixating the center of targets and the edge of obstacles.

## Methods

This section is organized into three parts. In the first part we review an analytical model that describes optic flow. In the second part we derive an analytical model for local spatial derivatives of optic flow for different edge types. In the third part we propose a method for the numerical computation of these analytical expressions. Procedures discussed in the latter two parts are then used to extract relevant properties of optic flow that are generated by an active observer following different strategies of gaze and heading control depending on the task.

### Models of Image Flow

#### Optic flow and image flow

Optic flow is defined as the apparent change of structured light in the optic array over time [2]. In contrast, we define image flow as the vector field that describes the displacement of 3D sample points projected onto the image plane due to either motion of the observer or of objects in the environment. Models of image motion have been suggested for translational and rotational instantaneous motion through a rigid environment for a passive observer [16] and an active observer maintaining fixed gaze [28], [33]. We next briefly recap these models for a passive and active observer and highlight their properties.

#### Image flow model for a passive observer

**Figure 9:**
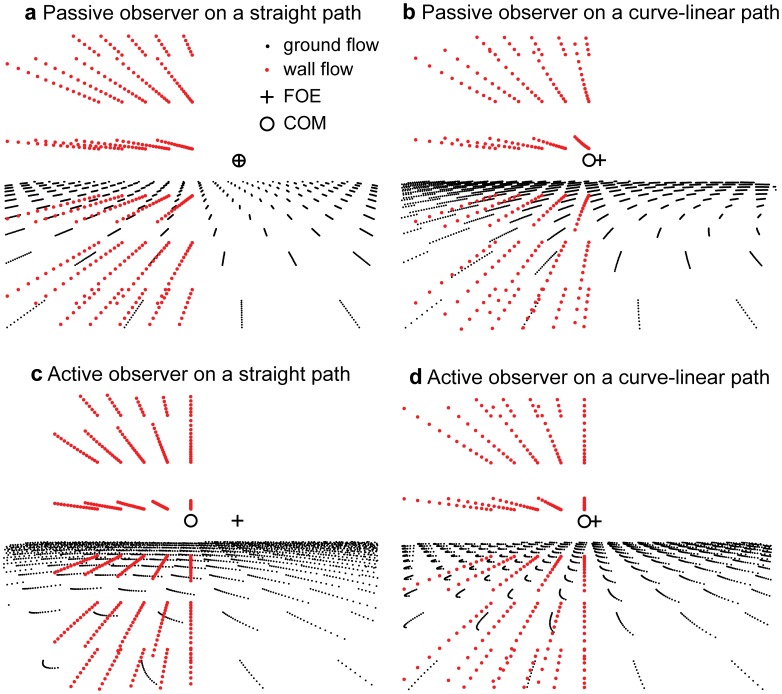
Display of image flows for a passive and an active observer. **a)** Translational flow for an observer moving along a straight path on a ground plane (black traces) and a pseudo-transparent object (red traces) that stands on this plane. The gaze is parallel with respect to the ground plane. The observer is passively sensing flow for the self-motion velocities 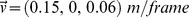 and 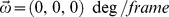. **b)** Curve-linear path for a passive observer with translational velocity 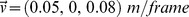 and rotational velocity 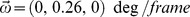. Note that in this case the vanishing point or center of motion (COM) is shifted away from the focus of expansion (FOE) and does not indicate the point being approached. **c)** The same configuration as in a); however, the observer is now actively maintaining gaze at the point 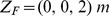. This fixation introduces a rotation around the *y*-axis (yaw rotation). Again FOE and COM do not coincide. **d)** The observer is on a curve-linear path while maintaining gaze at the point 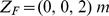. Note that the difference from c) is that the sample points are viewed different due to the additional body rotation. Active gaze control leads to strong differences of flow between foreground and background, in this example nearly 90°, compare a) with c) and b) with d). The legend in panel a) pertains to all panels.

For a model of optic flow we assume that an observer is moving passively through the environment without deploying head or eye movements. The observer’s body motion is defined as the linear velocity 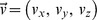 and the rotational velocity 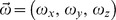, in classical mechanics this motion type is known as instantaneous motion [57]. One model of optic flow, called the image flow model, describes the projected temporal differential displacement 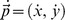 or pixel speed at the image location 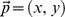 and this image location corresponds to the sample point 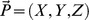 in the environment. For a pinhole camera model with the focal length 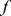 these two points are related by 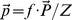. If the observer is moving the differential displacements on the image plane are given by Longuet-Higgins & Prazdny [16]:
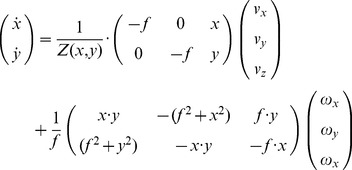
(1)In this model the heading vector is 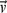 and the gaze vector is 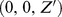, where the global coordinate system is defined by X′, Y′, and Z′ and an arbitrary sample point in this coordinate frame by X, Y, and Z. This image flow model has several important properties illustrated in Figure 9. Flow induced by translational motion is the translational flow – the first term on the right-hand-side – and flow induced by rotational motion is the rotational flow – the second term on the right-hand-side. Both flows superimpose linearly. Second, only the translational flow depends on the depth 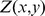 of the sample point in the scene that is accessible by “looking” or casting a ray through the image location 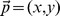. This depth dependency can be used to reconstruct relative depth from translational flow or to decide about nearness and farness of object surfaces visible to the camera. Absolute depth cannot be reconstructed because of the scaling invariance between the depth values and the translational speed; see also Equation (1). In Figure 9a nearer to the image plane (smaller Z) move faster than far points. Third, the rotational flow is independent of depth since the term 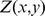 has been cancelled out in the components of the second term of the right-hand-side in Equation (1). Fourth, for a narrow field of view, i.e. a large focal length *f*, the rotational flow is barrel-shaped for a rotation around the *x*-axis (pitch rotation) or *y*-axis (yaw rotation). Fifth, translational forward motion leads to an expansion flow field with the focus of expansion (FOE) as point of source where flow vectors emerge. For backward motion the source becomes a sink where flow vectors vanish, called focus of contraction (FOC). Assuming a constant self-motion the FOE indicates the point the observer steers toward and eventually will approach this point in the scene. Sixth, if the translational motion is superimposed by pitch or yaw rotation the FOE or FOC is deflected, most importantly the vanishing point(s) in the flow, now called center of motion (COM), does not indicate the point the observer is steering toward, see Figure 9b. Seventh, the rotation around the *z*-axis (roll rotation) leads to a concentric flow with the center of rotation (COR) in the center of the image. All these properties help to describe the optic flow that is sensed by the eyes and to better understand its processing by the visual system. To facilitate this understanding we will further constrain the image flow model by introducing a fixating eye rotation (vestibule-ocular reflex) that is performed when humans maintain gaze fixed at a point in the environment while moving their body [58].

**Figure 10:**
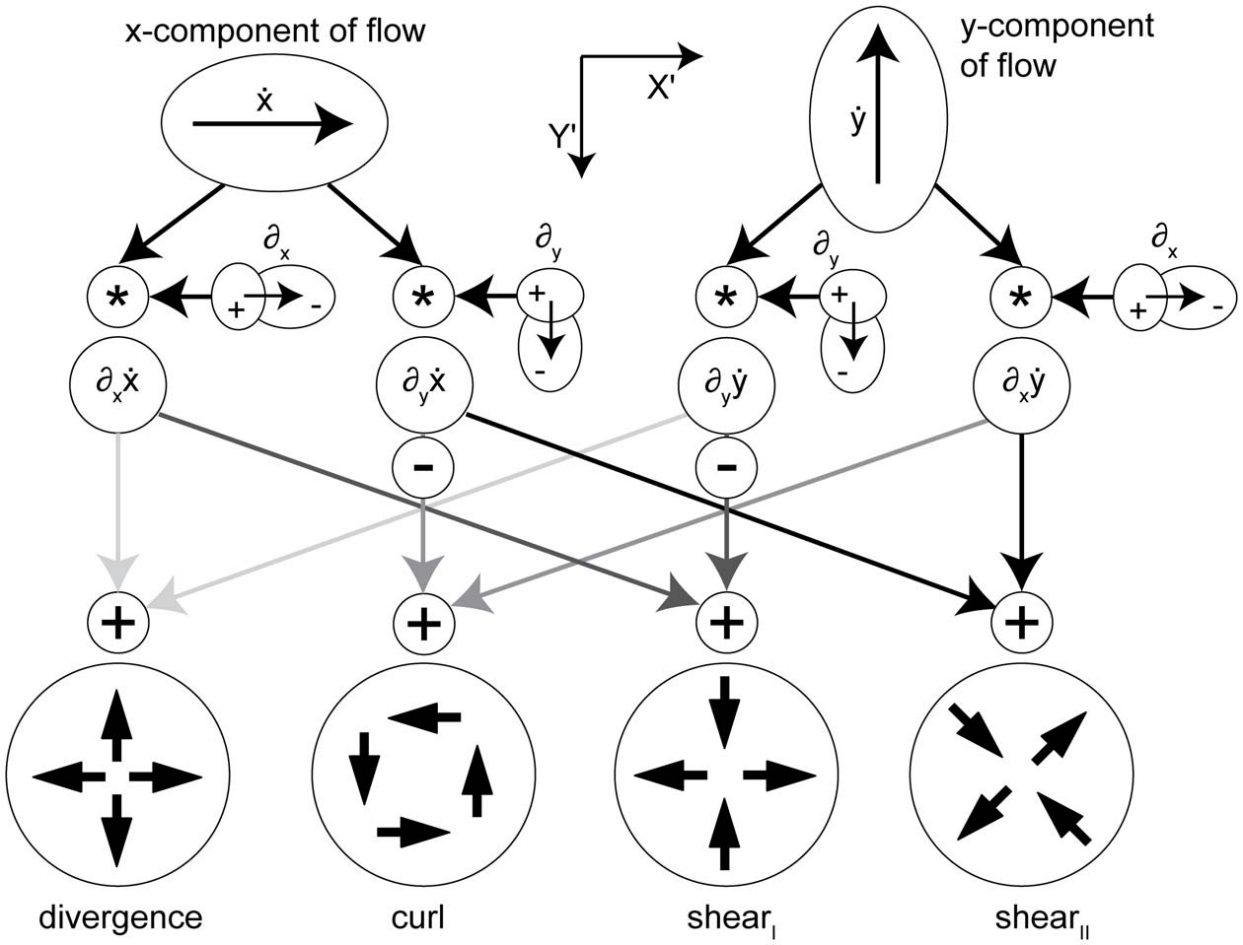
Shows a neural implementation of derivative operators and a network for the computation of divergence, curl, and shear. Derivative operators 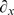 and 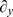 are applied to the flow components 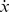 and 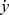. This gives the four entries of the Jacobian matrix, see Equation (5). Sums and differences between two entries of the Jacobian matrix result in divergence, curl, and shear components (last row). The symbol ‘*’ denotes the correlation with respect to the coordinate system shown in the top, middle of the figure.

#### Image flow model for an active observer maintaining gaze fixed

We call this observer active because he or she controls gaze by employing fixating eye-rotations in order to compensate for translational and rotational body motions. In order to maintain the point 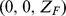 fixated and, thus, stationary on the image plane, pitch- and yaw-rotations are sufficient for an observer to perform if he is not trying to keep horizontal lines horizontal. Thus, we set roll-rotation to zero. Then, the model equation for fixating self-motion is [33], [59]:


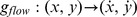
, with
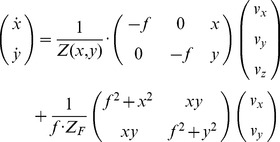
(2)Again, 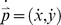 denotes the temporal change of sample points that appear at 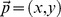 on the image plane using a pinhole camera model with the focal length *f*. The fixated point is 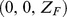 with the depth *Z_F_* measured along the optical axis or direction of gaze. On the right hand side of Equation (2) a linear superposition of two terms appears. The first term describes the translational flow that depends on 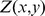, while the second term describes the rotational flow that occurs during the fixation and that is independent of 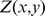. In comparison to Equation (1) the rotational velocities are now constrained by the translational velocities 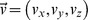 and the fixation point 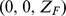. Note that Equation (2) excludes body or head rotations to simplify the model analysis. An example for a transformation of these rotations into the eye’s coordinate frame has been given by Waxman and Duncan [60].

The image flow model for a fixating rotation has some additional properties compared to the general model given in Equation (1). First, the COM, i.e. the point that appears stationary in the projection, is always at the location of the fixation point. This COM does not coincide with the FOE if gaze and heading point in different directions. Thus, the COM does not indicate the future point being approached, see also Figure 9c. Second, if the fixation point is very far, that is if 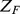 is large, the second summand in Equation (2) could be neglected. In other words, the flow becomes a pure translational flow. Third, there is an overall symmetry between the *x*- and *y*-component; consistently swapping of *x* and *y* yields the same expressions.

### Model of Spatial Flow-derivatives

An analytical model provides insights about the characteristics of flow derivatives under different conditions and informs the observer about objects in the scene. Furthermore, we constrain our model for the case of an edge to characterize the flow discontinuity of an edge.

#### Decompose spatially local flow derivatives

To study local, spatial flow derivatives using Equation (2) we compute the partial derivatives for each flow component with respect to the Cartesian *xy*-coordinate system. The four partial derivatives are summarized in the Jacobian matrix:
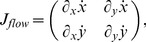
(3)where 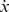 and 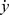 denote the image flow from Equation (1) or (2). These derivatives quantify the strength of flow change across the main coordinate axes. Note, the derivatives defined by Equation (3) do not directly denote acceleration or deceleration in the physical sense, which are defined as second-order temporal derivatives 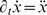 and 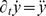. Derivatives of a large value indicate a discontinuity in the flow; either because of the depth discontinuity between foreground and background or because of independently moving objects in the visual field.

In the following analysis we begin with the flow from Equation (2). Plugging in the definition and computing the above partial derivatives results in the Jacobian matrix:
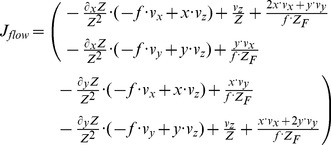
(4)


Note that we omitted the dependency of *Z* on the variables *x* and *y* in the notation in this Equation (4). No partial derivatives for *Z_F_* occur, since this value does not vary with the image coordinates, unlike to *Z*. The overall change in flow can be characterized by the decomposition into the four components of divergence, curl, Type I shear, and Type II shear:
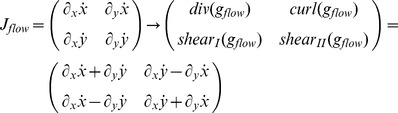
(5)The symbol ‘→’ denotes a coordinate transform into the new basis system defined by divergence, curl, and two shear components. This decomposition has been studied in psychophysics to find a basis system for motion perception [17], [18] and in neurophysiology to characterize the selectivity of cells [61], [62]. Table 2 summarizes the decomposition of flow derivatives into these terms and their interpretation is as follows: (i) Expanding and contracting flows relate to a positive and negative divergence or a source and sink, respectively, see Figure 2b. (ii) Clockwise and counterclockwise rotating flows correspond to negative and positive curl, respectively, see Figure 2c. (iii) Shear components express an expansion in one axis and a contraction in the orthogonal axis. For Type I shear both axes are aligned with the Cartesian *xy*-coordinate system, see Figure 2d. Type II shear references a 45° counterclockwise rotated Cartesian *xy*-coordinate system, see Figure 2e.

#### Scene model of a plane

To relate these general terms for divergence, curl, and shear to a behaviorally relevant scenario we further assume *Z(x,y)* to be defined by a plane
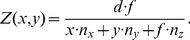
(6)This plane is defined by the normal vector 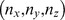 with 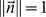 and the distance *d* that is measured along the normal vector; *x* and *y* denote the image coordinates, and *f* is again the focal length of the pinhole camera model. For instance, a normal vector of 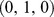 denotes a ground plane or ceiling and a normal vector of 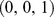 a back- or front-plane. The latter planes are parallel to gaze. Note only translational flow depends on this planar depth surface. Thus we only report divergence, curl, and shear in Table 1 for translational flow in combination with a plane.

To formalize the scenario of fixating an edge versus fixating the center of a planar surface we define a parametric plane with the normal 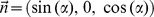 and the distance *d*. Note that a back-plane or front-plane is given by 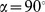. A decomposition of the Jacobian matrix for this case is given in Table 3 in the second row, where the *y*-component has been set to zero in order to simplify expressions. To formalize the fixation of an edge we apply the following trick. The back or front-plane is rotated by 90°, see also Figure 4b. For the observer then it appears as a single line assuming that this plane is fixated in the center of the visual field. Thus, fixation of an edge can be expressed as the limit case 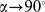 and we identify the distance of the plane by the horizontal image coordinate 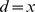 to account for the case if the plane does not appear exactly in the center of the visual field. The result of this approximation is shown in the second to last row of Table 3. These flow components express the flow derivative components for looking at a planar surface.

#### Scene model of a cylinder

An edge can also be defined as looking tangentially onto a circle with the radius *R* that appears at the distance *d* measured along the optical axis and at *R* to the right. In order to simplify calculations we assume a parallel projection for this circle, thus, *x*  =  *X*, and assume *x* to be small which is the case for foveal vision. This gives the depth values:
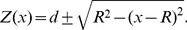
(7)By computing the partial derivatives for *x* and *y* and plugging the result into the equations in second row of Table 2, all last row entries in Table 3 are derived. These last row entries describe the decomposition of flow components for a smoothly curved surface that has been modeled by a cylinder.

#### Scene model of an apical edge

The derivation of a scene model of an apical edge, example in Figure 1, is given in the following steps. First, we describe the edge by a line. This line is then projected onto a ground plane and could be interpreted as a shadow line beaming light from the camera’s position. Second, both lines are rotated according to the camera’s orientation. Third, differences in depth between corresponding points on the two lines are computed. Fourth, these differences are assumed to approximate the partial derivative of the depth function in the *x*-dimension. The partial derivative in the *y*-dimension is assumed to be approximately zero.

Assume that the edge line is given as 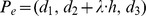 with the starting position 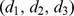, height 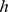, and 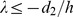. In our case 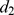 is negative because the line starts below the camera’s position that is 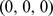. The ground plane is defined by 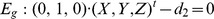 with the normal vector pointing up in the direction of the *y*-axis and the distance 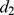 below the camera. Projecting the edge line onto the ground results in the shadow line 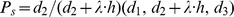. Both lines are rotated by applying the rotation matrix 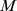. This gives the rotated points 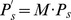 and 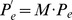. The rotated version of the lines’ dots is indicated by an apostrophe. Computing the difference between the rotated lines results in:
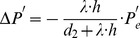
(8)Thus, we approximate the partial derivatives of the function 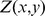 by the following expressions:
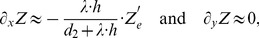
(9)with 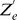 denoting the depth of the fixated edge. This depth is plugged into Equation (4) for all instances where 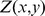 occurs. These two terms are plugged into the definition of divergence, curl, and shear components from Table 2. This gives the following expressions for an apical edge:



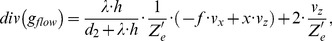
(10a)

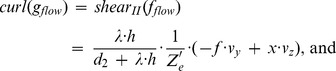
(10b)

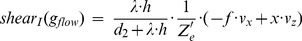
(10c)


Note that in order to match the definition for pixel flow that is used in the simulations the Equation (10b) has to be multiplied by minus one, since the y-axis is considered to be negative in order to point downward for image pixel coordinates. When the image coordinates 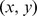 are close to gaze, 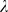 approaches 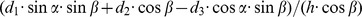. More general, image coordinates that fall onto the edge line are:
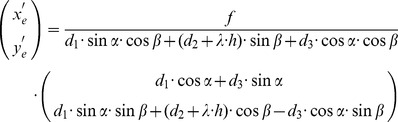
(11)The angle 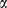 denotes a rotation around the *y*-axis, the angle 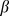 a rotation around the already rotated *x*'-axis, and 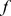 the focal length of the pinhole camera model. This finishes the derivation of flow derivatives for an apical edge and the change in flow between the foreground object and the ground in the back.

### Simulation of Divergence, Curl, and Shear Responses for Synthetic Video Sequences

#### Specification of scene and computation of analytical flow

The scene is defined by a ground plane with the normal vector 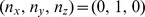 and the distance 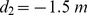. This defines the camera to be 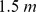 above the ground. The focal length of the simulated pinhole camera is 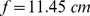 (or 2748.45 pixels) assuming a display size of 1 cm×1 cm or 240×240 pixels. This definition corresponds to a visual field of five degrees horizontally and vertically. The post’s height is 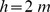 and its width and depth are 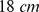, respectively. For target approach this post appears with its center at the position 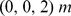. That is two meters away measured along the optical axis. The translational velocity of the camera is 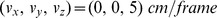. For obstacle avoidance the translational velocity is 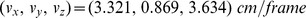, the post appears at the position 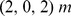, and the fixation point is 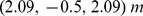. For this scene images were rendered using the ray-tracer from Persistence of Vision Pty. Ltd. [63]. This allows us to compute the depth map 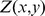 and, thus, analytical flow by using the above Equation (2).

#### Computation of flow derivatives

Partial derivatives 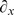 and 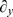 of the flow are approximated using biologically inspired operators with antagonistic and asymmetric center-surround [22]. These operators are similar to the motion-opponent operators used for the estimation of self-motion and relative depth [64] as well as the estimation of self-motion in combination with a monopole mapping [35]. We define these biologically inspired operators by using the difference of two Gaussian subfields. For the partial derivative in the x-component the positive Gaussian subfield that models the positive center of a cell’s receptive field has a standard deviation of two pixels in the y-component and one pixel in the x-component. This models an elliptical shape for the Gaussian kernel. The kernel’s size is 9×5 pixels. The negative Gaussian subfield or negative surround of a cell’s receptive field has a standard deviation of two pixels in the y-component and three pixels in the x-component and its modeled size is 9×13 pixels. To align correlation results from the two subfields we shift the center result by one pixel to the left and the surround result by two pixels to the right. See also the icon for x-derivatives in Figure 4. This assumes the negative subfield to be on the right and the positive subfield to be on the left. For the partial derivative in the y-component this circuit is 90° rotated in counterclockwise direction, see also the icon for y-derivatives in Figure 10. Then the decomposition into divergence, curl, and, shear components is calculated by using the Equation (5), compare also with the circuit shown in Figure 10. The x- and y-flow components are correlated with the Gaussian subfields that are subtracted in order to compute the partial x- and y-derivatives for each flow component. This results in four combinations that are shown in the middle of the circuit in Figure 10. Sums and differences of these four partial derivatives result in divergence, curl, and shear components. See bottom row in Figure 10.

## Supporting Information

Appendix S1 **Contains the deviation of flow derivatives for a monopole mapping that models cortical flow.** The spatial-flow derivatives in form of divergence, curl, and shear have large values (±infinity in the limit) at depth discontinuities in the fovea and periphery with the exception of curl and shear Type II components being zero-valued in the periphery.
(PDF)
