# Supplementary material for: Active Gaze Control Improves Optic Flow-Based Segmentation and Steering
Source: PLoS One. 2012 Jun 14;7(6):e38446. doi: 10.1371/journal.pone.0038446 (PMC3375264; doi:10.1371/journal.pone.0038446)
Supplement: Appendix S1 — Contains the deviation of flow derivatives for a monopole mapping that models cortical flow. The spatial-flow derivatives in form of divergence, curl, and shear have large values (±infinity in the limit) at depth discontinuities in the fovea and periphery with the exception of curl and shear Type II components being zero-valued in the periphery. (PDF) [file pone.0038446.s001.pdf]

Raudies F, Mingolla E, Neumann H Active Gaze Control Improves Optic Flow-Based Segmentation and Steering. *PLoS One* (in press)

Supporting Information

Analytical models of spatial flow-derivatives including a monopole transform

So far, the analysis accounts for the central visual field of a few degrees of visual angle in cortical space. The cortical magnification within this few degrees of visual field can be approximated by a straight line. In order to find a formulation for peripheral flow the monopole (Schwartz, 1980), dipole, or wedge dipole (Balasubramanian et al., 2002) transform provide closed form analytical descriptions to account for cortical magnification. These transforms provide a mapping of visual field coordinates into coordinates of the visual cortex. In the following we give a model of derivatives for peripheral flow considering the monopole transform. Generally, a transform f_{trans} adds terms to the flow derivatives due to the chain rule, here, applied to the transformed flow:

$$g_{trans,flow}' = (g_{trans}(g_{flow}))' = g_{trans}' \cdot g_{flow} + g_{trans} \cdot g_{flow}'. \qquad (12)$$

The symbol ' denotes the total derivative of the transform and g_{flow} the function from Equation (2) that maps to every location x and y on the image plane their respective flow components $\dot{x}$ and $\dot{y}$. In our case we assume this transform to be given by the monopole transform defined as (Schwartz, 1977):

$$g_{mpole} : (x,y) \rightarrow w = \begin{cases} \log(z+a) - \log(a) & \text{if} \quad \mathrm{Re}(x) \geq 0 \\ -(\log(-z+a) - \log(a)) & \text{if} \quad \mathrm{Re}(x) < 0 \end{cases} \quad \text{with} \qquad (13)$$

$$z = x + i \cdot y \in C$$

This transforms uses the complex logarithm; z and w are complex numbers and i denotes the complex unit. For small values of a coordinates are mainly changed along

radial directions and less along angular directions. This corresponds to early observations about the mapping of visual space to cortex (Daniel & Whitteridge, 1961). The parameter $a > 0$ is added to avoid the singularity of the logarithm at zero.

In order to simplify the notation in the following deviations we restrict the calculations to the case $\mathrm{Re}(x) \geq 0$. Calculations for $\mathrm{Re}(x) < 0$ are analogous. The total derivative of the monopole transform is given by the Jacobian matrix (Elder et al., 2009; part of their Equation 4):

$$J_{mpole} = \frac{1}{(x+a)^2 + y^2} \begin{pmatrix} x+y & +y \\ -y & x+a \end{pmatrix} \quad \mathit{for} \quad \mathrm{Re}(x) \geq 0. \qquad (14)$$

This matrix can be computed as the partial derivatives of the mapping in Equation (13) with respect to the Cartesian coordinates *x* and *y* and then extracting the real part for the first-column entries and the imaginary part for the second-column entries of the matrix J_{mpole}. In the following we plug Equation (14) into the derivative of the transformation g_{trans}' in Equation (12). Then the derivative $g_{trans,flow}'$ for the monopole transform is given by:

$$\begin{aligned} J_{mpole,flow} = & \frac{1}{((x+a)^2 + y^2)^2} \begin{pmatrix} y^2 - (x+a)^2 & -2\cdot(x+a)\cdot y \\ -2\cdot(x+a)\cdot y & y^2 - (x+a)^2 \end{pmatrix} \cdot \begin{pmatrix} \dot{x} & \dot{y} \\ \dot{y} & \dot{x} \end{pmatrix} \\ & + \frac{1}{(x+a)^2 + y^2} \begin{pmatrix} x+a & y \\ -y & x+a \end{pmatrix} \cdot J_{flow} \quad \mathit{for} \quad \mathrm{Re}(x) \geq 0 \end{aligned}. \qquad (15)$$

In this Equation (15) the variables $\dot{x}$ and $\dot{y}$ refer to those in Equation (2). Plugging-in these variables into Equation (15) results in long expressions of rational polynomials with arguments in *x* and *y*. Fortunately, these expressions are simplified by using the definition of polar coordinates $x = r \cdot \cos(\varphi)$ and $y = r \cdot \cos(\varphi)$, where *r* denotes the radial distance from the center of the image plane and φ the angle with respect to the horizontal axis. In order to study the peripheral flow we compute the limit for *r* toward infinity. For the central flow we compute the limit *r* toward zero. Components of divergence, curl, and

shear for these limit cases are given in Table 5 for the monopole transform. The components in Table 5 are computed by using the partial derivatives ∂_x and ∂_y that are defined with respect to the Cartesian coordinates in the 2D image plane. This computation could be termed as an external view. Derivatives are computed with respect to the image plane. For an internal view derivatives could be defined with respect to a polar coordinate system using the partial derivatives ∂_r and ∂_φ. These latter definitions have often been used as a basis system to study neural selectivity in cortex, e.g. cortical magnification (Dow et al., 1981).

More details about the derivative components of the monopole transformed flow are provided by plugging in the definition of a plane from Equation (6). Results are shown in Table 6. In the periphery curl and type two shear vanish, see the upper half of Table 6. Divergence and type one shear components are independent of the angle φ, and can approach infinity in the limit if gaze is tangential to the fixated planar or curved surface, see upper half of Table 6. Flow derivatives for the fovea are given in the lower half of Table 6. Flow derivative components have a hyperbolic curve with respect to the argument d or x that is the distance of the planar or curved surface from the center of the visual field if these surfaces are tangentially fixated.

Periphery (r →∞)

	$div(g_{mpole,flow})$	$curl(g_{mpole,flow})$	$shear_I(g_{mpole,flow})$	$shear_{II}(g_{mpole,flow})$
Translation	$-\frac{\partial_x Z}{Z^2}\cdot v_z$	$+\frac{\partial_y Z}{Z^2}\cdot v_z$	$-\frac{\partial_x Z}{Z^2}\cdot v_z$	$-\frac{\partial_y Z}{Z^2}\cdot v_z$
Fixating rotation	$+\frac{1}{f\cdot Z_F}\cdot v_x$	$-\frac{1}{f\cdot Z_F}\cdot v_y$	$+\frac{1}{f\cdot Z_F}\cdot v_x$	$+\frac{1}{f\cdot Z_F}\cdot v_y$
Translation toward a plane	$-\frac{n_x}{e\cdot f}\cdot v_z$	$+\frac{n_y}{e\cdot f}\cdot v_z$	$-\frac{n_x}{e\cdot f}\cdot v_z$	$-\frac{n_y}{e\cdot f}\cdot v_z$

Fovea (r→0)

	$div(g_{mpole,flow})$	$curl(g_{mpole,flow})$	$shear_I(g_{mpole,flow})$	$shear_{II}(g_{mpole,flow})$
Translation	$2\frac{a\cdot v_z + f\cdot v_x}{a^2\cdot Z} + f\frac{\partial_y Z\cdot v_y + \partial_x Z\cdot v_x}{a\cdot Z^2}$	$2\frac{f\cdot v_y}{a^2\cdot Z} - f\frac{\partial_y Z\cdot v_x - \partial_x Z\cdot v_y}{a\cdot Z^2}$	$-f\frac{\partial_y Z\cdot v_y - \partial_x Z\cdot v_x}{a\cdot Z^2}$	$+f\frac{\partial_y Z\cdot v_x + \partial_x Z\cdot v_y}{a\cdot Z^2}$
Fixating rotation	$-\frac{2\cdot f}{a^2\cdot Z_F}\cdot v_x$	$-\frac{2\cdot f}{a^2\cdot Z_F}\cdot v_y$	0	0
Translation toward a plane	$2\frac{x\cdot n_x + y\cdot n_y + f\cdot n_z}{a\cdot e\cdot f}v_z + 2\frac{x\cdot n_x + y\cdot n_y + f\cdot n_z}{a^2\cdot e}v_x + \frac{n_y\cdot v_y + n_x\cdot v_x}{a\cdot e}$	$2\frac{x\cdot n_x + y\cdot n_y + f\cdot n_z}{a^2\cdot e}\cdot v_y - \frac{n_y\cdot v_x - n_x\cdot v_y}{a\cdot e}$	$-\frac{n_y\cdot v_y - n_x\cdot v_x}{a\cdot e}$	$+\frac{n_y\cdot v_x + n_x\cdot v_y}{a\cdot e}$

Table 5 Decomposition of the Jacobian $J_{mpole,flow}$ of the monopole transformed flow into divergence, curl, type one shear, and type two shear. All entries in this table are for the case x ≥ 0.

Periphery (r →∞)

	$div(g_{mpole,flow})$	$curl(g_{mpole,flow})$	$shear_I(g_{mpole,flow})$	$shear_{II}(g_{mpole,flow})$
Plane tilted with respect to gaze	$-\frac{\sin\alpha}{d\cdot f}\cdot v_z$	0	$-\frac{\sin\alpha}{d\cdot f}\cdot v_z$	0
Plane parallel to gaze [1]	$-\frac{1}{x\cdot f}\cdot v_z$	0	$-\frac{1}{x\cdot f}\cdot v_z$	0
Curved edge [2]	$-\frac{x}{D\cdot(z_0-D)^2}\cdot v_z$	0	$-\frac{x}{D\cdot(z_0-D)^2}\cdot v_z$	0

Fovea (r→0)

	$div(g_{mpole,flow})$	$curl(g_{mpole,flow})$	$shear_I(g_{mpole,flow})$	$shear_{II}(g_{mpole,flow})$
Plane tilted with respect to gaze	$2\frac{x\sin\alpha+f\cos\alpha}{a\cdot d\cdot f}\cdot v_z$ $+2\frac{x\sin\alpha+f\cos\alpha}{a^2\cdot d}\cdot v_x$ $+\frac{\sin\alpha}{a\cdot d}\cdot v_x$	$2\frac{x\sin\alpha+f\cos\alpha}{a^2\cdot d}\cdot v_y$ $+2\frac{\sin\alpha}{a\cdot d}\cdot v_y$	$+\frac{\sin\alpha}{a\cdot d}\cdot v_x$	$+\frac{\sin\alpha}{a\cdot d}\cdot v_y$
Plane parallel to gaze [1]	$+\frac{2}{a\cdot f}\cdot v_z+(\frac{2}{a^2}+\frac{1}{a\cdot x})\cdot v_x$	$+(\frac{2}{a^2}+\frac{1}{a\cdot x})\cdot v_y$	$+\frac{1}{a\cdot x}\cdot v_x$	$+\frac{1}{a\cdot x}\cdot v_y$
Curved edge [2]	$2\frac{a\cdot v_z+f\cdot v_x}{a^2\cdot(z_0-D)}$ $+f\frac{x\cdot v_x}{a\cdot D\cdot(z_0-D)^2}$	$2\frac{f\cdot v_y}{a^2\cdot(z_0-D)}$ $+f\frac{x\cdot v_y}{a\cdot D\cdot(z_0-D)^2}$	$+f\frac{x\cdot v_x}{a\cdot D\cdot(z_0-D)^2}$	$+f\frac{x\cdot v_y}{a\cdot D\cdot(z_0-D)^2}$

Table 6 Entries for the decomposition of flow derivatives for fixation of the center or edge of an obstacle that is modeled as a planar or circular surface. All entries in this table refer to the case x ≥ 0. [1]This assumes the normal $\vec{n}=(1,0,0)$. [2]The discriminant is $D=\sqrt{R^2-(x-x_0)^2}$.

References

Balasubramanian, M., Polimeni, J.R., and Schwartz, E.L. (2002). The V1-V2-V3 complex: quasiconformal dipole maps in primate striate and extrastriate cortex. *Neural Networks* 15, 1157-1163.

Daniel, P.M., Whitteridge, D. (1961). The representation of the visual field on the cerebral cortex in monkeys. *Journal of Physiology* 159, 203-221.

Dow, B.M., Snyder, A.Z., Vautin, R.G., and Bauer, R. (1981). Magnification factor and receptive field size in foveal striate cortex of the monkey. *Experimental Brain Research* 44, 213-228.

Elder, D.M., Grossberg, S., and Mingolla, E. (2009). A neural model of visually guided steering, obstacle avoidance, and route selection. *Journal of Experimental Psychology* 35(5), 1501-1531.

Schwartz, E. L. (1980). Computational anatomy and functional architecture of striate cortex: a spatial mapping approach to perceptual coding. *Vision Research* 20(8), 645-669.

Schwartz, E.L. (1977). Spatial mapping in the primate sensory projection: analytic structure and relevance to perception. *Biological Cybernetics* 25(4):181-194.
